# Infection, inflammation and intervention: mechanistic modelling of epithelial cells in COVID-19

**DOI:** 10.1098/rsif.2020.0950

**Published:** 2021-02-17

**Authors:** Nabil T. Fadai, Rahil Sachak-Patwa, Helen M. Byrne, Philip K. Maini, Mona Bafadhel, Dan V. Nicolau

**Affiliations:** ^1^ School of Mathematical Sciences, University of Nottingham, Nottingham NG7 2RD, UK; ^2^ Mathematical Institute, University of Oxford, Oxford OX2 6GG, UK; ^3^ Respiratory Medicine Unit, Nuffield Department of Medicine, University of Oxford, Old Road Campus, Oxford OX3 7LF, UK; ^4^ School of Mathematical Sciences, Queensland University of Technology, Brisbane, Queensland 4001, Australia

**Keywords:** hyperinflammation, inhaled corticosteroids, cytokine storm, COVID-19

## Abstract

While the pathological mechanisms in COVID-19 illness are still poorly understood, it is increasingly clear that high levels of pro-inflammatory mediators play a major role in clinical deterioration in patients with severe disease. Current evidence points to a hyperinflammatory state as the driver of respiratory compromise in severe COVID-19 disease, with a clinical trajectory resembling acute respiratory distress syndrome, but how this ‘runaway train’ inflammatory response emerges and is maintained is not known. Here, we present the first mathematical model of lung hyperinflammation due to SARS-CoV-2 infection. This model is based on a network of purported mechanistic and physiological pathways linking together five distinct biochemical species involved in the inflammatory response. Simulations of our model give rise to distinct qualitative classes of COVID-19 patients: (i) individuals who naturally clear the virus, (ii) asymptomatic carriers and (iii–v) individuals who develop a case of mild, moderate, or severe illness. These findings, supported by a comprehensive sensitivity analysis, point to potential therapeutic interventions to prevent the emergence of hyperinflammation. Specifically, we suggest that early intervention with a locally acting anti-inflammatory agent (such as inhaled corticosteroids) may effectively blockade the pathological hyperinflammatory reaction as it emerges.

## 1. Introduction

SARS-CoV-2, the causative viral agent for COVID-19 illness, has infected tens of millions of people and caused many deaths worldwide. As SARS-CoV-2 is a novel coronavirus, with ensuing COVID-19 a new illness, knowledge transfer has been piecemeal and only preliminary data exist for SARS-CoV-2 [1]. Treatment thus far has focused on severe COVID-19 [2] or the production of vaccines to prevent transmission [3,4]. However, as it has been recognized that COVID-19 produces a ‘runaway’ train pattern of hyperinflammation [5,6], it can be postulated that early intervention with an anti-inflammatory, such as inhaled corticosteroids (ICS), could contain the illness [7]. This hypothesis is supported further by the clinical finding that chronic respiratory diseases (such as asthma and chronic obstructive pulmonary disease, COPD) are not the most common co-morbidities seen in COVID-19 [8], indicating that ICS, a common treatment for asthma and COPD, could provide a protective response.

**Figure 1.**
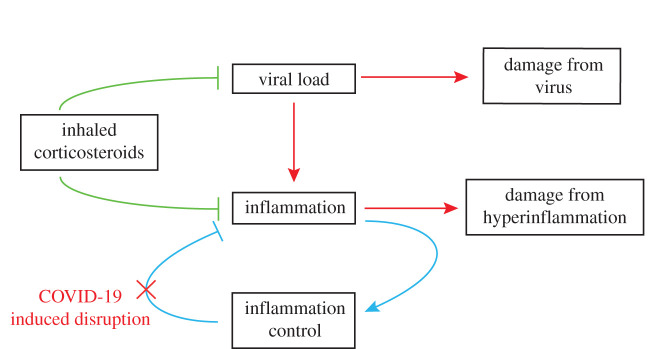
A simplified model of the potential inflammatory process in COVID-19 and potential therapeutic intervention. A rising viral load causes rising inflammation, which causes damage but is normally kept in check by a feedback mechanism. In COVID-19 and other respiratory infections, this mechanism appears to fail, leading, in a minority of patients, to a ‘runaway train’ pattern of hyperinflammation. Separately, viral entry into the vasculature leads to direct damage in the lung and in distal organs. Potential intervention early on with an anti-inflammatory agent targeted at lung epithelial cells may help to re-establish this check on hyperinflammation.

The majority of COVID-19 patients develop a respiratory illness [9], with the primary site of infection being the airway epithelium where the ACE2 receptors, necessary for SARS-CoV-2 infection [10], are in abundance. Furthermore, the emerging literature now contains evidence [7] that supports a conceptual model (figure 1) in which a viral infection triggers an immune response, which, in a minority of patients, is an immune-driven hyperinflammatory process [5], leading to an acute respiratory distress-like syndrome, as well as causing direct damage by other (e.g. vascular) means [11].

As we gain further understanding of SARS-CoV-2, it is clear that mechanistic investigations alone may not be sufficient and that mathematical models of a complex biological system are needed [12]. Specifically, mathematical models describing lung epithelial inflammation in COVID-19 may provide insight not accessible by experimental work alone [12]. Such approaches have previously been used to investigate the onset and resolution of inflammation [13,14], identify key parameters in the inflammatory process and better understand the interplay between inflammation and cell migration in epithelial tissue [15]. The urgent need better to understand COVID-19 pathology, combined with the necessarily partial and piecemeal experimental evidence emerging from clinics and laboratories, makes mathematical modelling a natural approach for hypothesis generation and testing, as well as designing treatment strategies.

We hypothesize that in at-risk patients, clinical deterioration is a combination of direct damage from the virus and a resulting uncontrolled pulmonary inflammatory process; we develop a mathematical model to explore these phenomena. In particular, we present a five-component mathematical model that describes inflammation kinetics, viral infection, and novel mechanisms that couple their dynamics. Using this model, we (i) investigate which qualitative differences in inflammation levels can be observed in patients infected by SARS-CoV-2, and (ii) determine how ICS treatment can be employed to reduce inflammation in the lung epithelium, thereby preventing the onset of hyperinflammation and other severe damage associated with high levels of pro-inflammatory mediators.

## 2. Results

**Figure 2.**
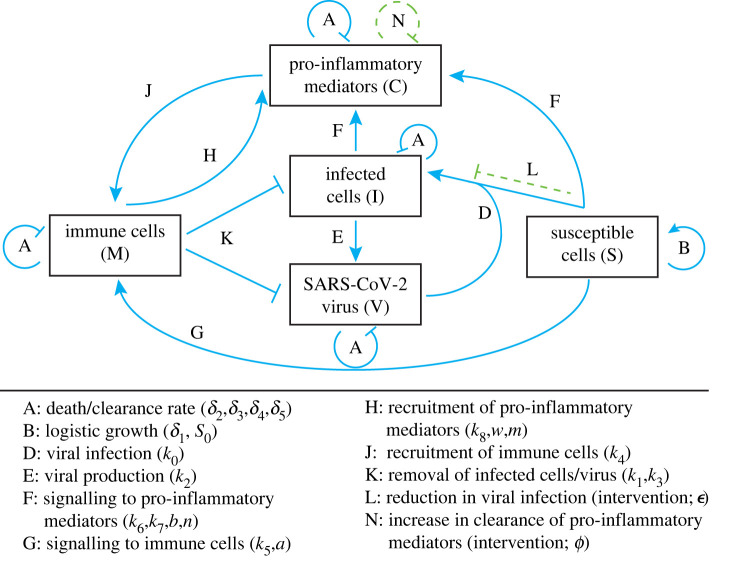
Network diagram describing the MVSIC model. Dimensional parameters are listed alongside each relevant process; processes resulting from intervention strategies are shown in dashed green.

Using the current understanding of COVID-19 [5,6], we consider a mathematical modelling framework that describes the temporal evolution of five species at early timescales of SARS-CoV-2 viral infection: pro-inflammatory mediators (e.g. cytokines, interferons, *C*), recruited immune system cells (e.g. lymphocytes, T-cells and macrophages, *M*), freely-moving SARS-CoV-2 virus (*V*), cells susceptible to viral infection (e.g. epithelial lung cells, *S*), and infected cells (*I*). We refer to this five-species mathematical model as the *MVSIC model*. These species interact via a network of physiological responses relating to the localized infection and inflammation of epithelial lung cells (see figure 2 for a model schematic). Unlike previous models describing inflammation or viral infection (c.f. [13,16–18]), the MVSIC model incorporates the novel additional feature of coupling inflammation kinetics to viral infection.

We assume that each species will have a natural death/clearance rate (process A in figure 2) and their dynamics are coupled via the following mechanisms. Firstly, we assume that susceptible epithelial lung cells follow logistic growth in the absence of infection (process B in figure 2) and become infected when free virus enters and reproduces inside it (process D in figure 2). Secondly, we assume that infected cells produce several copies of free virus; these new viruses leave the infected cell without destroying it (process E in figure 2). Thirdly, we assume that susceptible and infected cells can both ‘signal’ for cytokine recruitment [5,6] after detecting free virus (process F in figure 2); the infected cell signal may be smaller or even repress the susceptible cell's signals. This cell signalling mechanism is represented by rate-limited kinetics [19], which assumes a sigmoidal Hill-like reaction term [20], multiplied by the amount of signalling cells present (see §2.1 for further details). Fourthly, we assume that susceptible cells can also directly recruit (local) immune cells, bypassing the cytokine pathway, via rate-limited kinetics (process G in figure 2). While this direct pathway allows faster recruitment of immune cells, there are fewer immune cells locally available. Therefore, we also assume that cytokines are recruited via immune system cells (process H in figure 2) and vice versa (process J in figure 2), creating a positive feedback loop. Finally, we assume that immune cells can consume infected epithelial cells via phagocytosis and remove SARS-CoV-2 virus (process K in figure 2).

We are not only interested in the underlying physiological mechanisms driving inflammation in epithelial lung cells, but also the effect of various *intervention strategies* that can reduce viral infection and/or severe cases of inflammation. Therefore, we also consider two additional pathways associated with therapeutic intervention (green dashed lines in figure 2). One intervention pathway is the reduction of viral infection of susceptible cells (process L in figure 2), as might appear in retroviral therapies or vaccines; this therapy is represented by a reduction in the viral infection rate. The other intervention pathway that we will consider is the accelerated clearance of pro-inflammatory cytokines by anti-inflammatories (process N in figure 2), such as ICS [21].

### 2.1. The MVSIC model

We now propose a mathematical formulation of the network diagram shown in figure 2 associated with our five-species model, while incorporating the various physiological mechanisms outlined in the previous section. The temporal evolution of the five species outlined in the MVSIC model is described using the following system of ordinary differential equations (ODEs):

2.1 $$\frac{dS}{dt}=\delta_{1}S\left(1-\frac{S}{S_{0}}\right)-k_{0}(1-\epsilon )VS,$$

2.2 $$\frac{dI}{dt}=k_{0}(1-\epsilon )VS-\delta_{2}I-k_{1}MI,$$

2.3 $$\frac{dV}{dt}=k_{2}I-k_{3}MV-\delta_{3}V-k_{0}(1-\epsilon )VS,$$

2.4 $$\frac{dM}{dt}=k_{4}C-\delta_{4}M+k_{5}S\left(\frac{V}{a+V}\right),$$

2.5 $$\begin{array}{r} \frac{dC}{dt}=-\delta_{5}(1+\phi )C+(k_{6}S+k_{7}I)\left(\frac{V^{n}}{b^{n}+V^{n}}\right)+k_{8}M\left(\frac{V^{m}}{w^{m}+V^{m}}\right). \end{array}$$

The aforementioned rate-limited mechanisms, associated with signalling and recruitment, are represented by the rational functions in *V* appearing in equations (2.4) and (2.5). All model variables and parameters appearing in this dimensional version of the MVSIC model are described in table 1. In particular, we note that our intervention parameters associated with reducing the viral infection rate and increasing the pro-inflammatory cytokine clearance rate are denoted as *ε* and *ϕ*, respectively.

**Table 1.** Summary of variables and dimensional parameters used in the MVSIC model. All parameters have units of species population, unless otherwise stated.

variable/parameter	biological interpretation	parameter	biological interpretation
*S*(*t*)	susceptible cell population	*δ*_1_	low-density reproduction rate of susceptible cells (s^−1^)
*I*(*t*)	infected cell population	*δ*_2_	infected cell clearance rate (s^−1^)
*V*(*t*)	viral load	*δ*_3_	viral clearance rate (s^−1^)
*M*(*t*)	recruited immune cell population	*δ*_4_	immune cell clearance rate (s^−1^)
*C*(*t*)	cytokine population	*δ*_5_	cytokine clearance rate (s^−1^)
*k*_0_	viral infection rate (s^−1^)	*S*_0_	healthy equilibrium population of susceptible cells
*k*_1_	phagocytosis rate (s^−1^)	*a*	rate-limited viral concentration (direct signalling)
*k*_2_	viral production rate (s^−1^)	*n*	Hill power coefficient (indirect signalling; dimensionless)
*k*_3_	viral clearance rate from immune response (s^−1^)	*b*	rate-limited viral concentration (indirect signalling)
*k*_4_	immune cell recruitment rate from cytokines (s^−1^)	*m*	Hill power coefficient (immune cell signalling; dimensionless)
*k*_5_	immune cell recruitment rate from direct cell signalling (s^−1^)	*w*	rate-limited viral concentration (immune cell signalling)
*k*_6_	cytokine recruitment rate from indirect cell signalling (s^−1^)	*ε*	relative factor of infection inhibition from ICS (dimensionless)
*k*_7_	cytokine recruitment rate from indirect infected cell signalling (s^−1^)	*ϕ*	relative factor of additional cytokine clearance from ICS (dimensionless)
*k*_8_	cytokine recruitment rate from immune cells (s^−1^)		

While there are a large number of (dimensional) parameters present in the MVSIC model, certain combinations of these parameters can exhibit the same qualitative features to one another if particular *non-dimensional* parameter groupings remain unchanged. As a result, we now examine a non-dimensionalized version of the MVSIC model to better understand how the qualitative features of the model vary with respect to these non-dimensional parameter groupings. By rescaling the dimensional variables as

2.6 $$\begin{array}{rl} & S=S_{0}\hat{S},\;I=S_{0}\hat{I},\;V=S_{0}\hat{V},\\ & \;C=\frac{S_{0}k_{6}}{\delta_{1}}\hat{C},\;M=\frac{S_{0}k_{4}k_{6}}{\delta_{1}\delta_{4}}\hat{M},\;t=\frac{\hat{t}}{\delta_{1}}, \end{array}$$

we obtain, after dropping hats, the non-dimensional system

2.7 $$\frac{dS}{dt}=S[1-S-\kappa (1-\epsilon )V]$$

2.8 $$\frac{dI}{dt}=\kappa (1-\epsilon )SV-\gamma_{1}I(\lambda_{1}M+1)$$

2.9 $$\frac{dV}{dt}=\zeta I-\kappa (1-\epsilon )SV-\gamma_{2}V(\lambda_{2}M+1),$$

2.10 $$\frac{dM}{dt}=\gamma_{3}(C-M)+\rho S\left(\frac{V}{\alpha +V}\right),$$

2.11 $$\;\frac{dC}{dt}=(S+\sigma I)\left(\frac{V^{n}}{\beta^{n}+V^{n}}\right)+\mu M\left(\frac{V^{m}}{\omega^{m}+V^{m}}\right)-\gamma_{4}(1+\phi )C,$$

where

2.12 $$\begin{array}{rl} & \gamma_{1}=\frac{\delta_{2}}{\delta_{1}},\;\gamma_{2}=\frac{\delta_{3}}{\delta_{1}},\;\gamma_{3}=\frac{\delta_{4}}{\delta_{1}},\;\gamma_{4}=\frac{\delta_{5}}{\delta_{1}},\;\lambda_{1}=\frac{S_{0}k_{1}k_{4}k_{6}}{\delta_{1}\delta_{2}\delta_{4}},\\ & \lambda_{2}=\frac{S_{0}k_{3}k_{4}k_{6}}{\delta_{1}\delta_{3}\delta_{4}},\;\kappa =\frac{S_{0}k_{0}}{\delta_{1}},\zeta =\frac{k_{2}}{\delta_{1}},\;\mu =\frac{k_{4}k_{8}}{\delta_{1}\delta_{4}},\;\rho =\frac{k_{5}\delta_{4}}{k_{4}k_{6}},\\ & \sigma =\frac{k_{7}}{k_{6}},\;\alpha =\frac{a}{S_{0}},\;\beta =\frac{b}{S_{0}},\;\omega =\frac{w}{S_{0}}. \end{array}$$

Since *δ*_1_, the growth rate of epithelial lung cells, typically evolves on the timescale of days [22], which is a similar timescale to early stages of SARS-CoV-2 infection, this is an appropriate choice of timescale to non-dimensionalize the MVSIC model. Finally, the non-dimensional MVSIC model (2.7)–(2.11) also includes initial conditions for the five species to reflect that initially, there is a small amount of virus present, no susceptible cells have been infected yet, and no inflammatory mediators or immune cells are present at the site of infection:

2.13 $$\begin{array}{rl} S(0)=1,\;I(0) & =0,\;V(0)=V_{0},\;M(0)=0,\\ & \;C(0)=0. \end{array}$$

For simplicity, and minimal loss of generality, we will assume from here onwards that both Hill power coefficients, *n* and *m*, are equal to 2. These power coefficients represent a slower uptake of recruitment at low concentrations, but a larger response at moderate and high concentrations [19,20]. Furthermore, this choice of *n*, *m* > 1 agrees with the model description of having a faster local recruitment for small viral loads, but is surpassed at moderate viral loads by cytokine-related signalling pathways for immune cell recruitment.

### 2.2. Qualitative features of the MVSIC model

**Figure 3.**
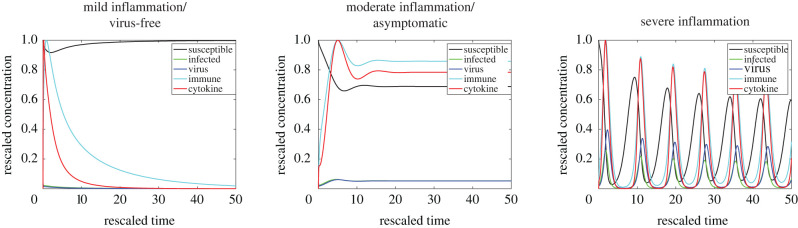
Qualitative states observed in the MVSIC model (2.7)–(2.13). The three different states (mild inflammation/virus-free state, asymptomatic/moderate inflammation, and severe inflammation) are characterized based on the levels of pro-inflammatory cytokines (red) present in the system, recruited by immune cells (cyan), as well as the quantity of susceptible cells (black) being infected (green) by virus (blue). Changing certain parameters causes the virus-free/mild state to transition to the asymptomatic/moderate state, or be further driven to the severe inflammation state. Parameter values used in the MVSIC model simulations are listed in table 2.

One of the objectives for developing the MVSIC model is to create a mechanistic model capable of describing five main clinical features observed in patients infected with SARS-CoV-2: (i) healthy patients that clear the virus without experiencing symptoms, (ii) asymptomatic patients that do not clear the virus but do not experience symptoms, (iii–v) patients experiencing mild, moderate, or severe levels of inflammation, the latter of which are also incapable of clearing the virus on their own, resulting in a hyperinflammatory state. These traits are not only based on the percentage of susceptible cells present, but also on the levels of pro-inflammatory mediators in the system. In other words, the virus-free and mild inflammatory states may look similar in terms of percentage of healthy epithelial cells, but are distinguished by the differing levels of pro-inflammatory mediators present. As such, we will define three main categories of qualitative features of the MVSIC model, based on percentage of healthy susceptible cells: (a) virus-free/mild inflammatory state, (b) asymptomatic/moderate inflammatory state, and (c) severe inflammatory state. Categories (a) and (b) are further subdivided between virus-free or mild inflammation (asymptomatic or moderate inflammation) based on the absolute magnitude of the cytokine levels. However, it is important to note that the *dimensional* quantity of pro-inflammatory mediators and immune cells present could indeed be large or small, depending on the parameters chosen in the rescaling of *M*(*t*) and *C*(*t*). As such, our analysis of the three infection scenarios is based on the *qualitative* features of the immunological responses, so *M*(*t*) and *C*(*t*) are rescaled in figure 3 by their maximal value without loss of generality.

**Table 2.** Dimensionless parameter groupings used in simulations of the MVSIC model.

qualitative state	parameters	state features
all	*γ*_3_ = 3, *γ*_4_ = 3, *λ*_1_ = 0.1, *λ*_2_ = 0.1, *ζ* = 8, *ρ* = 0.5, *σ* = 0.1, *μ* = 1, *n* = 2, *m* = 2, *α* = 0.05, *β* = 0.1, *ω* = 0.2, *V*_0_ = 0.05, *ε* = 0, *ϕ* = 0	
mild/virus-free	*γ*_1_ = 3, *γ*_2_ = 3, *κ* = 8	stable virus-free steady state
moderate/asymptomatic	*γ*_1_ = 4, *γ*_2_ = 4, *κ* = 6	stable infectious steady state
severe inflammation	*γ*_1_ = 5, *γ*_2_ = 5, *κ* = 8	unstable infectious steady state

To demonstrate that the MVSIC model is capable of producing all of these situations, we examine the solutions of the MVSIC model (2.7)–(2.13) for various choices of parameter values. Solutions are numerically computed in Matlab via the function ode15s and illustrative example solutions are shown in figure 3. Crucially, we are able to simulate all qualitative states of viral infection, characterized via the amount of cytokines present *C*(*t*) and percentages of susceptible cells *S*(*t*), using different parameter values in the MVSIC model. Furthermore, by performing an extensive sensitivity analysis of various dimensionless parameter groupings (electronic supplementary material), we can identify which pathways have the strongest connections to severe cases of inflammation. We use the extended Fourier amplitude sensitivity analysis (eFAST) [23,24], a variance decomposition technique, which measures the relative contributions of each individual parameter as well as the contributions of parameter interactions on key model outputs that serve as proxies for qualitative features observed in the hyperinflammatory state. We consider three model outputs: the minimum number of susceptible cells (electronic supplementary material), the average fluctuations in cytokine levels (electronic supplementary material), and the difference in maximum and minimum cytokine levels after a certain amount of time has passed (e.g. *t* > 20; electronic supplementary material). This sensitivity analysis demonstrates that the parameter groupings associated with viral infection and cytokine clearance (i.e. *γ*_1_, *γ*_2_, *γ*_4_, *κ* and *ζ*) most significantly affect moderate and severe cases of inflammation. In other words, despite lacking precise parameter values appearing in the MVSIC model, as might be obtained from experiments, the salient features of the MVSIC model exist in large regions of parameter space and are most significantly characterized by a small number of parameter groupings.

Mathematically, there are two key features that distinguish these three qualitative states: the absence or presence of an *infectious steady state*, and if this infectious steady state is stable or unstable. When the infectious steady state does not exist, the system settles to the *healthy steady state*, whereby the virus is cleared by the immune system and susceptible cells are replenished to their full quantities (*S* = 1). We will now examine additional stability features of these equilibria.

### 2.3. Equilibria and stability

A question that naturally arises from the MVSIC model is what qualitative features do we expect to observe for a given set of parameter values. To answer this question, we examine the existence and stability of various steady states, or equilibria, present in the MVSIC model. A steady state is a combination of constant values (S*, I*, V*, M*, C*) for which the right hand sides of (2.7)–(2.11) are all zero. From (2.8) and (2.9), any equilibria of the MVSIC model must have

2.14 $$I^{*}=\frac{\kappa (1-\epsilon )}{\zeta }S^{*}V^{*}+\frac{\gamma_{2}}{\zeta }V^{*}(\lambda_{2}M^{*}+1)=\frac{\kappa (1-\epsilon )S^{*}V^{*}}{\gamma_{1}(\lambda_{1}M^{*}+1)}.$$

Furthermore, from (2.7), we either have S* = 0 or S* = 1 − *κ*(1 − *ε*)V*, the former corresponding to the case when susceptible cells are depleted. If S* = 0, then we have from (2.8) that I* = V* = M* = C* = 0; we will refer to this equilibrium as the *zero state*, which can be shown to always be unstable. Alternatively, if S* = 1 − *κ*(1 − *ε*)V*, then (V*, M*, C*) satisfy the following nonlinear system of equations:

2.15 $$\begin{array}{rl} & \kappa (1-\epsilon )V^{*}[1-\kappa (1-\epsilon )V^{*}][\zeta -\gamma_{1}(\lambda_{1}M^{*}+1)]\\ & \;=\gamma_{1}\gamma_{2}V^{*}(\lambda_{1}M^{*}+1)(\lambda_{2}M^{*}+1), \end{array}$$

2.16 $$C^{*}=M^{*}-\frac{\rho V^{*}[1-\kappa (1-\epsilon )V^{*}]}{\gamma_{3}(\alpha +V^{*})},$$

2.17 $$\begin{array}{rl} & \gamma_{4}(1+\phi )C^{*}-\mu M^{*}\left(\frac{V^{*m}}{\omega^{m}+V^{*m}}\right)\\ & \;=[1-\kappa (1-\epsilon )V^{*}]\left[1+\frac{\sigma \kappa (1-\epsilon )V^{*}}{\gamma_{1}(\lambda_{1}M^{*}+1)}\right]\left(\frac{V^{*n}}{\beta^{n}+V^{*n}}\right). \end{array}$$

Notably, if V* = 0 in (2.15), this implies that M* = C* = I* = 0 and S* = 1; this equilibrium represents the *healthy steady state*. Otherwise, we obtain a new equilibrium in which V*, M*, C*, I*, S* are all strictly positive; we refer to this equilibrium as the *infectious steady state*. Furthermore, it can be shown (electronic supplementary material) that the infectious steady state can only exist when

2.18 $$\gamma_{1}<\frac{\zeta \kappa (1-\epsilon )}{\gamma_{2}+\kappa (1-\epsilon )}.$$

In other words, when susceptible cells become infected faster than infected cells being cleared from the system and the amount of free virus is sustained above a threshold quantity, the system settles to an infectious steady state. It can be shown that this infectious steady state, when (2.18) holds, is unique (electronic supplementary material). Additionally, the healthy steady state is unstable only when (2.18) holds, as this inequality corresponds to when all the eigenvalues of the Jacobian of (2.7)–(2.11) at the healthy steady state no longer have negative real parts. Therefore, the system can only clear the virus and inflammation responses completely if and only if the infectious steady state does not exist.

**Figure 4.**
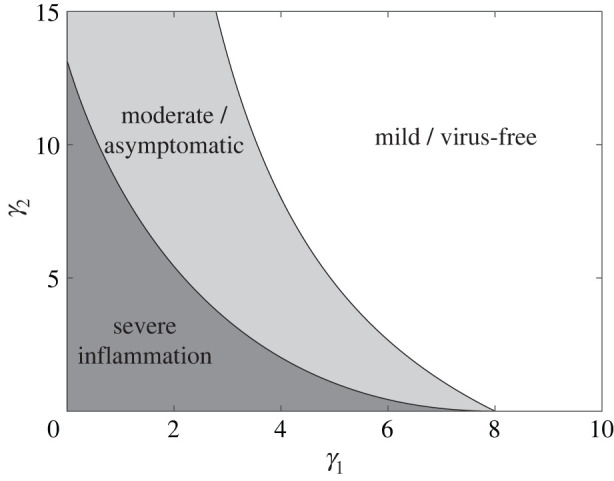
Bifurcation diagram of qualitative states in the MVSIC model. The mild inflammation parameter regime in table 2 is used while varying *γ*_1_ and *γ*_2_. The inequality (2.18) corresponds to the light and dark grey regions of (*γ*_1_, *γ*_2_) space. The infectious steady-state undergoes a Hopf bifurcation at the boundary of the light and dark grey regions, whereby the infectious steady state becomes unstable (dark grey).

In addition to the existence of a unique infectious steady state, we can also numerically compute where this infectious steady state becomes unstable. As shown in figure 4, the infectious steady state undergoes a Hopf bifurcation in parameter space, whereby two of the eigenvalues of the Jacobian of (2.7)–(2.11) at the infectious steady-state transition from having a negative real part to having a positive real part. This transition causes the infectious steady state to be locally unstable and, as seen in the severe inflammation state in figure 3, exhibit large oscillations in all species concentrations.

We also note that the ICS intervention parameter associated with pro-inflammatory cytokine clearance, *ϕ*, does not appear in (2.18). While increasing cytokine clearance cannot *remove* the infectious steady state, we will show in the next section that this parameter is linked to a reduction in the pro-inflammatory mediators, thereby eliminating a potential hyperinflammatory response.

### 2.4. Intervention strategies

We now examine how two key pathways in the MVSIC model, related to the viral infection rate and the pro-inflammatory cytokine clearance rate, are altered in the presence of a medical intervention, such as ICS. One intervention strategy is to reduce the rate at which susceptible cells become infected, as might be achieved by a vaccine. In the MVSIC model, this therapy corresponds to the parameter *ε*, which does indeed drive severe cases of inflammation down to the virus-free state as *ε* increases, as shown via the inequality (2.18) . However, in the absence of a vaccine option for SARS-CoV-2, we must also consider other treatment strategies that reduce inflammation, even if it does not completely eliminate the virus from a patient.

**Figure 5.**
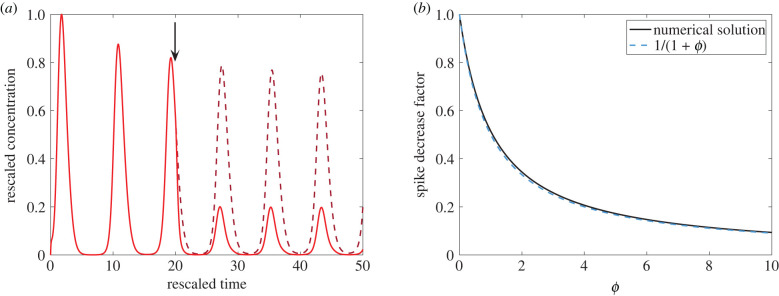
Intervening inflammation using inhaled corticosteroids. The severe inflammation parameter regime in table 2 is used prior to intervention (0 < *t* < 20). (*a*) At *t* = 20 (black arrow), *ϕ* is increased from 0 to 3 (red solid curve), reducing pro-inflammatory cytokine levels. The *C*(*t*) trajectory without intervention (*ϕ* = 0) is shown as a dark red dashed curve. (*b*) The height of the pro-inflammatory cytokine spike, relative to no intervention, decreases as *ϕ* increases. The numerically computed spike height decrease is shown in solid black, while the approximate decrease factor *F*(*ϕ*) = 1/(1 + *ϕ*) is shown in dashed blue.

Another intervention strategy is to increase the clearance rate of pro-inflammatory cytokines, thereby reducing the amount of inflammation present near infected cells. Since inhaled corticosteroids (ICS) act directly on epithelial lung cells, this therapy corresponds to increasing the parameter *ϕ* without any time delay. While ICS intervention cannot *remove* the infectious steady state, it can reduce large fluctuations of pro-inflammatory cytokines and the resulting damage that occurs in cases of severe inflammation. In figure 5*a*, we observe that the pro-inflammatory cytokine levels in the severe inflammation parameter regime (table 2) are reduced to about 25% when *ϕ* is increased from 0 to 3. Therefore, even in the case where ICS do not decrease the viral infection rate, they are still able to significantly reduce inflammation and augment the natural clearance rate of pro-inflammatory mediators. Indeed, by varying *ϕ* in magnitude, we see in figure 5*b* that the height of the pro-inflammatory cytokine spike decreases as *ϕ* increases. Relative to no intervention, i.e. *ϕ* = 0, we note that the spike's height decreases approximately by the relative factor *F*(*ϕ*) = 1/(1 + *ϕ*), which agrees with the numerical solution of the spike decrease factor with high accuracy (figure 5*b*). In other words, if ICS augment the natural cytokine clearance rate by a multiplicative factor of 1 + *ϕ*, then the height of cytokine spikes are *reduced* by approximately 1/(1 + *ϕ*) (e.g. doubling the effective clearance rate halves the spike height).

## 3. Discussion

We have developed the first mathematical model describing the dynamics of inflammation arising in epithelial lung cells infected with SARS-CoV-2. This model, named the MVSIC model, incorporates a network of mechanistic and physiological pathways that link together five distinct species, along with pathways associated with therapeutic interventions. We determine that the MVSIC model gives rise to distinct qualitative classes of COVID-19 patients: (i) individuals who naturally clear the virus, (ii) asymptomatic carriers and (iii–v) individuals who develop a case of mild, moderate, or severe illness as characterized by levels of inflammatory mediators.

The pathological mechanisms in COVID-19 illness are still being elucidated and very much an active topic of investigation. However, it is recognized that patients infected with SARS-CoV-2 have high levels of pro-inflammatory mediators [25], especially those that have severe illness. In the lung itself, pro-inflammatory monocyte-derived macrophages are abundant in the bronchoalveolar lavage fluid from patients with severe COVID-19 [26]. These, and other, findings point to a hyperinflammatory state in severely ill patients, which is believed to be linked to poor or fatal outcome, with a clinical trajectory that resembles acute respiratory distress syndrome (ARDS). Therefore, understanding, and preventing, dangerously high levels of inflammatory mediators present in patients with COVID-19 would appear to be crucial. Our model is consistent with these immunological findings; furthermore, this analysis points to potential therapeutic interventions to prevent the emergence of hyperinflammation (e.g. UK clinical trial NCT04416399 [7]). Specifically, we suggest that an early intervention with a locally acting (i.e. targeted at inflamed epithelial lung cells) anti-inflammatory agent may effectively lead to blockade in the ‘runaway train’ inflammatory reaction. Inhaled corticosteroids or other cytokine-reducing medications, as recently suggested [7], are candidates for such agents.

Our model is based on a simplified picture of the inflammatory pathways involved and therefore has limitations. It does not account comprehensively for every mechanism and cell class involved in SARS-CoV-2 infections and damage. We do not, for instance, distinguish between T-cells, macrophages, lymphocytes, and other immune cell types, instead focussing on the inflammatory mechanism as a whole. Additionally, in severe cases of COVID-19, recent evidence indicates that the virus permeates into the endothelium tissue of the lungs, circulating through the blood and causing distal organ damage by vascular and other means [27], while here we do not consider this direct/non-inflammatory avenue of viral damage. The timescale associated with SARS-CoV-2 permeating into the endothelium is far longer than local epithelial infection kinetics. Thus, the MVSIC model dynamics are relevant chiefly during the earlier stages of COVID-19 illness, when inflammatory processes dominate the clinical picture.

Finally, while certain immune responses, such as phagocytosis, produce inflammatory mediators, other immune responses, such as T-cells removing virus, do not. Thus, more detailed mathematical models are likely to be needed to account for the complex interplay between differing classes of immune cells; however, the MVSIC model is a first attempt to understand the biological complexity, which will require prospective validation. Enhancing our knowledge using these mathematical models is expected to be valuable in designing more sophisticated and patient-specific intervention strategies.

## Data accessibility

All Matlab codes used to generate figures 3–5 are available at https://github.com/nfadai/MVSIC2020
